# Naked Oat (*Avena nuda* L.) Oligopeptides: Immunomodulatory Effects on Innate and Adaptive Immunity in Mice via Cytokine Secretion, Antibody Production, and Th Cells Stimulation

**DOI:** 10.3390/nu11040927

**Published:** 2019-04-24

**Authors:** Ruixue Mao, Lan Wu, Na Zhu, Xinran Liu, Rui Liu, Yong Li

**Affiliations:** 1 Department of Nutrition and Food Hygiene, School of Public Health, Peking University, Beijing 100191, China; maoruixue@163.com (R.M.); summer920503@163.com (N.Z.); liuhappy07@163.com (X.L.); liuruipku@163.com (R.L.); 2 Inner Mongolia Administration for Market Regulation, Hohhot 010010, China; wulan_5@163.com

**Keywords:** oat, immunomodulatory oligopeptide, cell-mediated immunity, humoral immunity, macrophage phagocytosis, NK cell activity, T cell subpopulation, cytokine, immunoglobulin

## Abstract

The study aimed to investigate the immunomodulatory activity of oligopeptides derived from oat (*Avena nuda* L.) (OOPs). Healthy female BALB/c mice were randomly assigned to five groups, given deionized water (control) and 0.25, 0.5, 1.0, and 2.0 g/kg body weight (BW) of OOPs daily by intragastric administration. Seven assays were performed to determine the immunomodulatory effects of OOPs on immune organ ratios, cellular and humoral immune responses, macrophage phagocytosis, and natural killer (NK) cell activity. Spleen T lymphocyte subpopulations (by flow cytometry), serum cytokine and immunoglobulin levels (by multiplex sandwich immunoassays) were determined to evaluate how OOPs affected the immune system. Our results showed that OOPs could significantly improve innate and adaptive immune responses in mice through the enhancement of cell-mediated and humoral immunity, macrophage phagocytosis capacity, and NK cell activity. We concluded that the immunomodulatory effects might be attributed to increased T and Th cell percentages, serum interferon (IFN)-γ, interleukin (IL)-1 α, IL-2, IL-6, IL-10, IL-12, tumor necrosis factor (TNF)- α, and granulocyte-macrophage colony-stimulating factor (GM-CSF) secretions as well as immunoglobulin (Ig) A, IgG, and IgM productions. These results indicate that dietary OOPs could be considered as promising immunomodulators with dosages ranging from 0.25 to 2.0 g/kg BW.

## 1. Introduction

With growing unhealthy lifestyles as well as the exposure to stress and environmental extremes, impacts on the immune system have drawn great attention [1]. The immune system is a network of cells, tissues, and organs, protecting humans from viruses, bacteria, fungi, the growth of cancer cells, etc. Several drugs and chemicals have been developed as immunostimulants to modulate human immunity; however, further investigations of other approaches are required due to their high cost and inevitable side effects [2]. Among all choices, nutritional interventions have raised great expectations for their effectiveness and wide applications to enhance innate and adaptive immunities as well as resistance to diseases. Various active ingredients derived from natural foods have been demonstrated to be safe and effective in immunity enhancement. Particularly, bioactive peptides derived from food proteins have been shown to be promising in immune modulation for their low-molecular weights, highly digestible and absorbable features, and immunomodulatory, antimicrobial, antioxidative, antithrombotic, antihypertensive, and cholesterol-lowering activity [3,4,5]. Since the biological activity of peptides is strongly associated with the protein origin (specific amino acid sequences and bioavailability), whereas immunomodulatory effects of plant-derived peptides have been less explored [6], the effects of plant-derived peptides on immunity improvement are well worth exploring. 

Oat (often referred to *Avena sativa* L.) as an excellent whole grain choice, has been widely used for centuries for its various nutrients and health benefits [7,8,9]. The composition and health benefit of naked oat (*Avena nuda* L.), one of the ancient crops originating from China, still requires further investigation, even though the historical references on its detailed applications can be traced back to 1000 years before common era [10]. In the past decades, studies have demonstrated that naked oat contains sizable nutrients even superior to common oats, such as the highest content of proteins (approximately 16%) among all grains, unsaturated fatty acids (approximately 7%), carbohydrates (approximately 62%), and soluble fiber (approximately 5%) [11,12]. Notably, oat protein is not only outstanding in total content, but also in its balanced amino acid composition and high-level of lysine, which most grains lack. On the other hand, the booming demand of oat products and oil has resulted in a great quantity of oat extraction residues, which are abundant in nutritional proteins (mostly albumin and globulin) with a protein efficiency ratio of more than 2.0 [13,14]. Except for their nutritional properties, studies on the health effects of oat proteins or peptides are still limited, only involving hyperglycemia, hypertension and dyslipidemia regulations, anti-fatigue effects, and celiac disease [15,16,17,18,19]. Worth mentioning, these studies mainly focused on oat proteins or polypeptides, which have distinctly different absorption mechanisms compared to oat oligopeptides [20]. Oat oligopeptides are more digestible and absorbable than polypeptides with much lower molecular weights and higher bioavailability. Even immunoregulatory effects of food-derived peptides can be of great importance, and naked oat-derived oligopeptides can be a promising immunomodulator superior to both oat proteins and polypeptides, the immunoregulatory effects of oat oligopeptides have not been observed. We aimed to investigate the immunomodulatory effects of oat (*Avena nuda* L.) oligopeptides (OOPs) by intragastric administration in BALB/c mice with deionized water treatment as a control, and then explored the possible mechanisms.

## 2. Materials and Methods 

### 2.1. OOPs Preparation

OOPs were derived from oat (*Avena nuda* L.) bran by enzymatic hydrolysis and obtained from Weoat Group AG (Inner Mongolia, China). Briefly, after oil extraction by supercritical extraction, oat bran was homogenized and centrifuged, then hydrolyzed by multiple proteases. After a series of processing including activated carbon-adsorption, nanofiltration, cryoconcentration, decolorization, purification, and spray drying, the OOPs sample was obtained. After being purified by high-performance liquid chromatography (HPLC, 600, Waters, USA), matrix-assisted laser desorption ionization time-of-flight mass spectrometry (MALDI-TOF-MS, TripleTOF 4600, AB Sciex, USA) was used to determine the content and molecular weight distribution of the OOPs sample which showed that the content of the OOPs was a mixture of small molecule oligopeptides, over 92.05% of which had relative molecular weight < 1000 Da. The automatic amino acid analyzer (H835, Hitachi, Japan) was used as well to analyze the amino acid composition along with the molecular weight distribution of OOPs (shown in Table 1). 

### 2.2. Animals and Treatment

Eight-week-old female BALB/c mice (18–22 g) were obtained from the Animal Service of Peking University Health Science Center (Beijing, China). All experimental procedures were approved by the Peking University Animal Research Committee (Laboratory animal production license number: SCXK (Jing) 2016-0010; Laboratory animal use license number: SYXK (Jing) 2016–0041), following the Guide for the Care and Use of Laboratory Animals (NIH publication no. 85–23, revised 1996). The mice were randomly divided into five groups: control group (deionized water) and four OOPs groups (0.25, 0.5, 1.0, and 2.0 g/kg BW) after one-week-acclimatization. All treatments were administrated by gavage for 30 days. The mice were maintained under constant conditions at a temperature of 23 ± 1 °C, a humidity of 40–60%, a photoperiod of 12 h, and were fed the AIN-93 diet. Every week, the food and water intake and body weight of the mice were measured. 

### 2.3. Sample Collection and Preparation

After a 30-day-intervention, blood samples were collected from the ophthalmic venous plexus of the mice, then centrifuged at 3000 rpm for 10 min to obtain the serum. 

As for the preparation of the spleen cell suspension, spleens of the mice were collected in Hank’s balanced salt solution (HBSS) under aseptic conditions and gently squashed then filtrated with 200-mesh stainless steel sieves. After centrifugation twice with HBSS, the obtained single cell suspension was resuspended in red blood cell lysis buffer for red blood cell removal. Then viability and density of splenocytes were assessed after centrifugation and resuspension.

### 2.4. Assessment Protocols of Immunomodulatory Activity

#### 2.4.1. Phagocytic Capacity Assessment of Peritoneal Macrophages on Chicken Red Blood Cell (CRBCs) Clearance

CRBCs obtained from White Leghorn cocks were used to immunize mice by intraperitoneal administration of 1 mL of 20% (*v*/*v*) defibrinated CRBCs. Fifty mice (n = 10 in each group) were sacrificed after 30 min, and the peritoneal cavity was lavaged with 2 mL of 0.9% saline solution. After 1 mL of peritoneal-cell-rich lavage fluid was smeared on glass slides, all slides were successively incubated for 30 min at 37 °C, washed with saline, fixed with acetone and methanol (1:1, *v*/*v*), and stained with 4% (*v*/*v*) Giemsa–PBS solution. The percentage of CRBCs-phagocytosed-macrophages per 100 macrophages was counted as the phagocytic rate, and the number of phagocytosed CRBCs per 100 macrophages was counted as the phagocytic index.

#### 2.4.2. The Carbon Clearance Test

The carbon clearance method was used to assess the phagocytic activity of the reticuloendothelial system (RES) by intravenous injection of 0.1 mL per 10 g BW of pre-warmed diluted India ink into the lateral tail vein of mice. A volume of 20 μL blood samples from 50 mice (10 mice per group) were collected at 2 and 10 min intervals from the ophthalmic venous plexus, and mixed with 2 mL of 0.1% sodium carbonate solution. Sample absorbance was measured at 600 nm. Meanwhile, these mice were sacrificed with spleens and livers being removed and weighed. The carbon clearance index [α, Δoptical density (OD)/time] was calculated using the following equations:

α = Body weight × 3√κ / (liver weight + spleen weight)

κ = (lg OD_1_ − lg OD_2_) / (t_2_ − t_1_)

where OD_1_ and OD_2_ are the optical densities at 2 and 10 min, respectively.

#### 2.4.3. Spleen Natural Killer (NK) Cell Activity Analysis

Splenic NK cell activity was determined using the lactic acid dehydrogenase (LDH) release method [21]. Briefly, splenocyte cells (2 × 10^6^ cells/mL) from 50 mice (10 mice in each group) and YAC-1 cells (4 × 10^4^ cells/mL) were incubated in 96-well plates for 4 h at an effector/target cell ratio of 50:1 as the test well, a mixture of medium and target cells as the spontaneous release well, and a mixture of 2.5% Triton and target cells as the maximum release well. After 5 min of centrifugation at 1500 rpm, LDH substrates were mixed with supernatants, then incubated for 10 min at 37 °C. Their absorbance at 490 nm was determined in an enzyme-linked immunosorbent assay (ELISA) reader, and NK cell activity (%) was analyzed as follows:

NK cell activity (%) = (OD_test_ − OD_spontaneous_) × 100 / (OD_maximum_ − OD_spontaneous_)

#### 2.4.4. Delayed-Type Hypersensitivity (DTH) Reaction, Immunoglobulin (Ig) M-Plaque-Forming Cell (IgM-PFC) Test, and Serum Hemolysin Level Measurement

On day 26, 50 mice (n = 10 per group) were immunized by intraperitoneal injection of 0.2 mL of 2% (*v*/*v*) defibrinated sheep red blood cells (SRBCs). On day 30, the left-rear-footpad-thicknesses of mice were measured with a vernier caliper as the baseline. Subsequently, the mice were given subcutaneous administration of 20 μL of 20% (*v*/*v*) defibrinated SRBCs into the left rear footpads. 24 h after, the thicknesses of the left rear footpads were calculated with the same vernier caliper, and the footpad thickness variations were counted as the degrees of swelling.

On day 31, the immunized mice were sacrificed and the spleen cell suspensions were adjusted to a concentration of 5 × 10^6^ cells/mL for the IgM-PFC assay by the modified Jerne’s method. In brief, 0.5 mL of 0.5 g/mL agar solution mixed with 20 μL of the splenocyte suspensions and 50 μL of 10% (*v*/*v*) defibrinated SRBCs were fully poured onto a slide, which was covered with a layer of 0.5% agarose. Slides were then incubated at 37 °C, 5% CO_2_, and adequately reacted with guinea pig complement. The quantity of produced plaques was counted and expressed as the PFC amount per 10^6^ splenocytes.

Serum half hemolysis values (HC_50_) were measured using mice serum samples. A volume of 0.5 mL of 10% (*v*/*v*) SRBCs and 1 mL of 1:10 diluted guinea pig complement were added successively to the sample tubes with (test) or without (control) 1 mL of 1:200 diluted mouse serum. After a 30 min reaction in a water bath at 37 °C, sample tubes were immersed in an ice bath to terminate the reaction, then centrifuged to collect supernatants (approximately 1 mL/sample). Each supernatant was added into 3 mL of Drabkin’s reagent (0.05 g KCN, 0.2 g K_3_Fe(CN)_6_, and 1.0 g NaHCO_3_ dissolved in 1 L of distilled water), meanwhile, 0.25 mL of 10% (*v*/*v*) SRBCs was added into 3.75 mL of Drabkin’s reagent as a positive control. The absorbance at 540 nm was determined after a 10 min reaction, and the HC_50_ was calculated as follows: HC_50_ = (OD_sample_ / OD_positive control_) × 200.

#### 2.4.5. Splenocyte proliferation assay

Spleen cell suspensions (5 × 10^6^ cells/mL) obtained from 50 mice (n = 10 for each group) were pipetted into 24-well plates (1 mL/well) with (test) or without (control) 75 μL/well concanavalin (Con) A, and incubated for 68 h at 37 °C in a humid atmosphere with 5% CO_2_. A volume of 0.7 mL of supernatant in each well was replaced with RPMI-1640 without FBS, then 50 μL of thiazolyl blue tetrazolium bromide (MTT, 5 mg/mL) was added to each well. After a 4 h incubation, 1 mL/well of sodium dodecyl sulfate (SDS) solution (3%, w/w) was added, and the absorbance at 570 nm was evaluated in an ELISA reader (Bio-Rad, CA, USA). The absorbance differences between with and without the presence of Con A represented the proliferation capacity of splenocytes.

#### 2.4.6. Splenic T Lymphocyte Subpopulation Assay 

The splenocyte suspensions (1 × 10^6^ cells/mL) obtained from 50 mice (10 mice per group) were labeled with PerCP-Cy5.5-conjugated anti-mouse cluster of differentiation (CD) 3, fluorescein isothiocyanate-conjugated anti-mouse CD4, phycoerythrin-conjugated anti-mouse CD25, and allophycocyanin-conjugated anti-mouse CD8a or isotype controls (all from BioLegend, CA, USA) at 4 °C in the dark for 30 min. The labeled lymphocytes were washed twice, resuspended in cell staining buffer (BioLegend, CA, USA), and analyzed by flow cytometry [22]. 

#### 2.4.7. Multiplex Sandwich Immunoassay of Cytokine and Immunoglobulin Levels

Serum cytokine and immunoglobulin levels were evaluated from the serum samples with a Milliplex mouse 11 cytokine/5 immunoglobulins premixed kit (Millipore, MA, USA) using the xMAP® platform (Luminex, TX, USA). Eleven cytokines and three immunoglobulins were determined according to the manufacturer’s instructions using the MAGPIX® instrument (Luminex, Texas, USA) to read, and Milliplex Analyst 5.1 software (Millipore, MA, USA) to analyze. 

### 2.5. Statistical Analysis

All values were expressed as mean ± standard deviation (SD). One-way analysis of variance (ANOVA) with Least Significant Difference (LSD) was used to evaluate the significance of differences among groups. A *p* value of < 0.05 was considered to be statistically significant.

## 3. Results

### 3.1. Effects of OOPs on Body Weight and Immune Organs

As shown in Table 2, by calculating the index of the spleen and thymus as spleen or thymus weight /body weight, we found that the daily intake of OOPs did not lead to any significant impact on the body weight and index of the spleen and thymus, nor on the food or water intake (*p* > 0.05), suggesting that OOPs were nontoxic to the treated mice.

### 3.2. Effects of OOPs on Humoral Immunity

**Figure 1:**
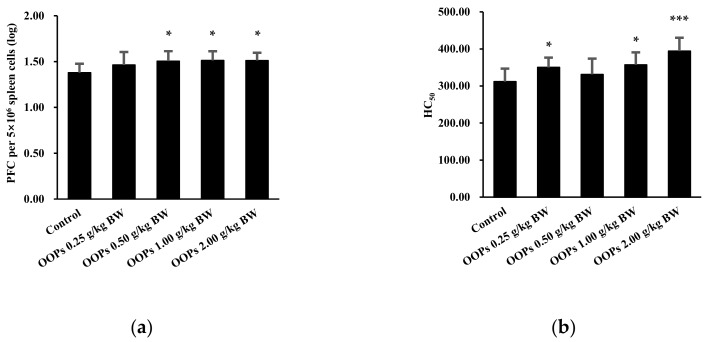
Effects of OOPs on humoral immunity in mice. (**a**) Spleen immunoglobulin (Ig) M antibody response to sheep red blood cells (SRBC); (**b**) Serum half hemolysis value (HC_50_). Data was represented as mean ± SD, *n* = 10. * *p* < 0.05 vs. control group, *** *p* < 0.001 vs. control group.

We assessed the effects of OOPs on humoral immunity in mice via the spleen IgM antibody response to SRBC and serum HC_50_ (Figure 1). Results of the IgM-PFC test showed that the PFC of 5 × 10^6^ spleen cells in OOPs 0.5, 1.0, and 2.0 g/kg BW groups were respectively increased by 9.14%, 9.70%, and 9.63% compared to the control group (*p* < 0.05, Figure 1a). As shown in Figure 1b, the HC_50_ in OOPs 0.25, 1.00, and 2.0 g/kg BW groups were significantly improved compared with that of the control group (*p* < 0.05, *p* < 0.05, and *p* < 0.001, respectively). 

### 3.3. Effects of OOPs on Cell-Mediated Immunity

**Figure 2:**
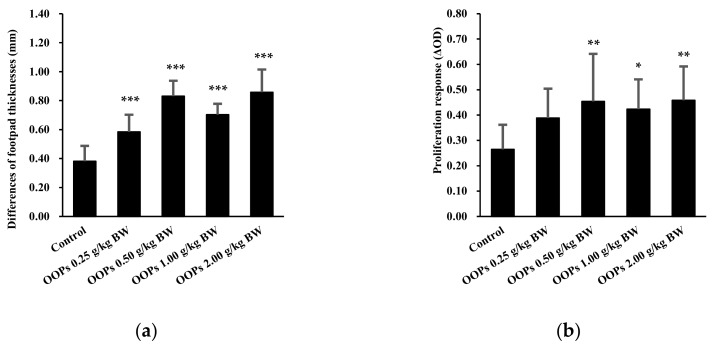
Effects of OOPs on cell-mediated immunity in mice. (**a**) Delayed-type hypersensitivity reaction to SRBCs; (**b**) ConA-stimulated splenic lymphocyte proliferation. Data was represented as mean ± SD, *n* = 10. * *p* < 0.05 vs. control group, ** *p* < 0.01 vs. control group, *** *p* < 0.001 vs. control group.

The results of the DTH reaction to SRBCs are presented in Figure 2. Compared with the control group, significant differences in mouse footpad thicknesses were observed in all four OOPs groups, increased by 53.13%, 117.80%, 84.33%, and 124.97%, respectively (*p* < 0.001, Figure 2a). Moreover, there was an increases in the ConA-stimulated splenic lymphocyte proliferation of mice in OOPs 0.5, 1.00, and 2.0 g/kg BW groups (*p* < 0.01, *p* < 0.05, and *p* < 0.01, respectively) and an increasing trend in the OOPs 0.25 g/kg BW group (*p* = 0.062) compared with the control group (Figure 2b). 

### 3.4. Effects of OOPs on Macrophage Phagocytosis

**Figure 3:**
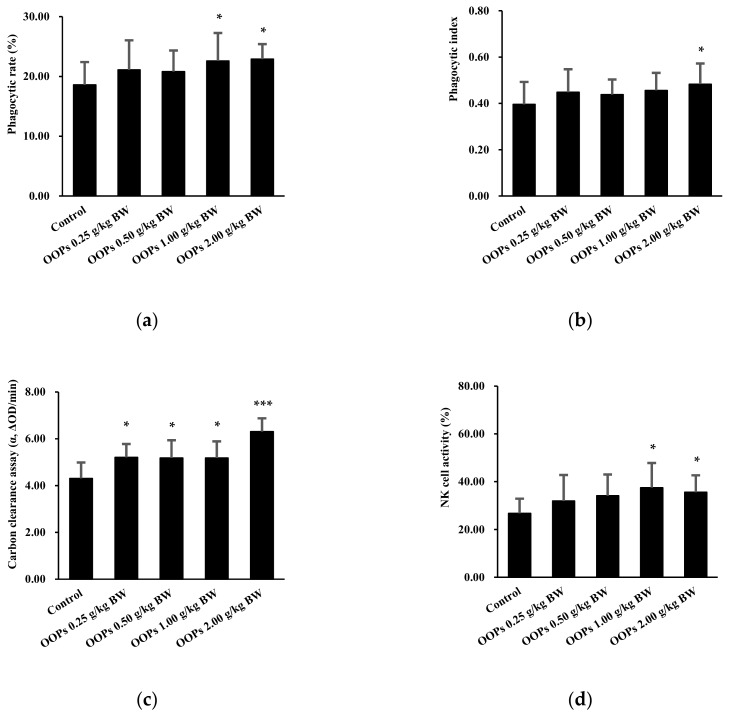
Effects of OOPs on macrophage phagocytosis capacity and NK cell activity in mice. (**a**) The phagocytic rate of peritoneal macrophage phagocytosis in CRBCs; (**b**) Phagocytic index of peritoneal macrophage phagocytosis in CRBCs; (**c**) Macrophage phagocytosis in carbon clearance; (**d**) NK cell activity. Data are represented as mean ± SD, *n* = 10. * *p* < 0.05 vs. control group, *** *p* < 0.001 vs. control group.

The phagocytic capacity of macrophages was assessed by macrophage phagocytosis both in CRBCs and carbon clearance capacity tests. Compared with that in the control group, we found that the phagocytic capacity of peritoneal macrophages in clearing CRBCs was significantly elevated, with respectively increased phagocytic rates (Figure 3a) of 21.51% and 23.12% in OOPs 1.0 and 2.0 g/kg BW groups, and a phagocytic index (Figure 3b) of 21.97% in the OOPs 2.0 g/kg BW group. As shown in Figure 3c, macrophage phagocytosis in the carbon clearance test was significantly improved by 20.88%, 20.28%, 20.23%, and 46.43% in the OOPs 0.25, 0.5, 1.0 g/kg BW groups (*p* < 0.05), and 2.0 g/kg BW group (*p* < 0.001) respectively compared with those in the control group. 

### 3.5. Effects of OOPs on NK Cell Activity

NK cell activity is also an important component in the formation of the innate immune system other than macrophage phagocytosis. We observed that NK cell activity in mice treated with OOPs 1.0 and 2.0 g/kg BW was enhanced significantly by 40.27% and 33.17%, respectively compared with that of the control treatment (*p* < 0.05, Figure 3d).

### 3.6. Effects of OOPs on Splenic T Lymphocyte Subpopulations

Since OOPs may potentially increase T cell activity via quantity alternation of T cells or their subpopulations, phenotypic analysis of T lymphocyte subpopulations was conducted. According to the results shown in Table 3, the percentage of total T cells (CD3^+^) was significantly increased in mice treated with OOPs 1.0 and 2.0 g/kg BW by 11.94% and 12.68% (*p* < 0.05 and *p* < 0.01, respectively). Moreover, the percentage of CD4^+^ CD8^−^ in OOPs 1.0 and 2.0 g/kg BW groups were elevated significantly in comparison with that in the control group (both *p* < 0.01). 

### 3.7. Effects of OOPs on Cytokine Concentrations in Serum

**Figure 4:**
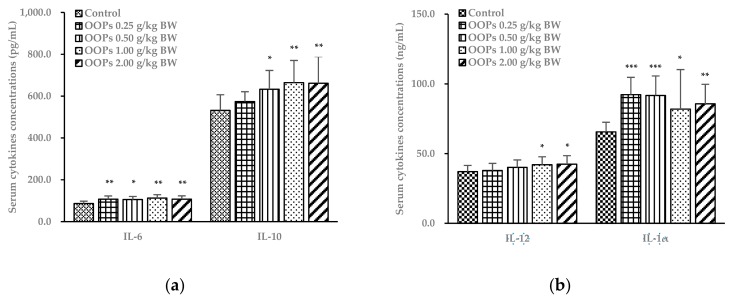

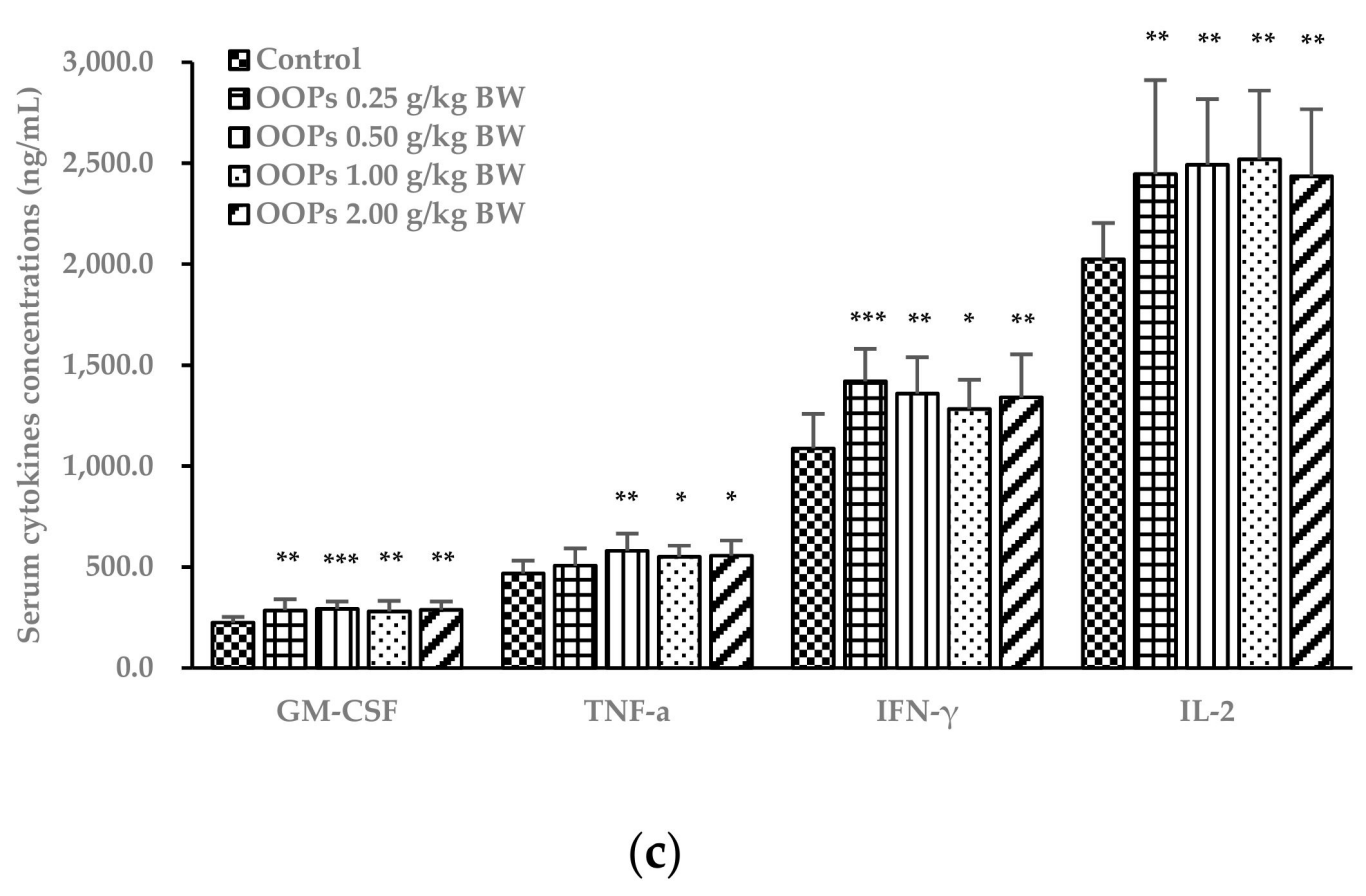
Effects of OOPs on cytokine concentrations in serum. (**a**) Interleukins (IL)-6 and IL-10 concentrations in serum; (**b**) IL-12 and IL-1α concentrations in serum; (**c**) granulocyte-macrophage colony-stimulating factor (GM-CSF), tumor necrosis factor-α (TNF-α), interferon-γ (IFN-γ) and IL-2 concentrations in serum. Data are represented as mean ± SD, *n* = 10. * *p* < 0.05 vs. control group, ** *p* < 0.01 vs. control group, *** *p* < 0.001 vs. control group.

As shown in Figure 4, serum interleukins (IL)-1α, IL-2, IL-6, interferon-γ (IFN-γ), and granulocyte-macrophage colony-stimulating factor (GM-CSF) levels in all OOPs groups were significantly increased (*p* < 0.05). In contrast to those in the control group, IL-10 and tumor necrosis factor-α (TNF-α) concentrations in OOPs 0.5, 1.0, and 2.0 g/kg BW groups were significantly increased as well (*p* < 0.05). In addition, mice in the OOPs 1.0 and 2.0 g/kg BW groups showed elevated IL-12 levels in serum. There were no significant differences in IL-4 and IL-5 concentrations among all groups (*p* > 0.05). 

### 3.8. Effects of OOPs on Serum Immunoglobulin Levels 

**Figure 5:**
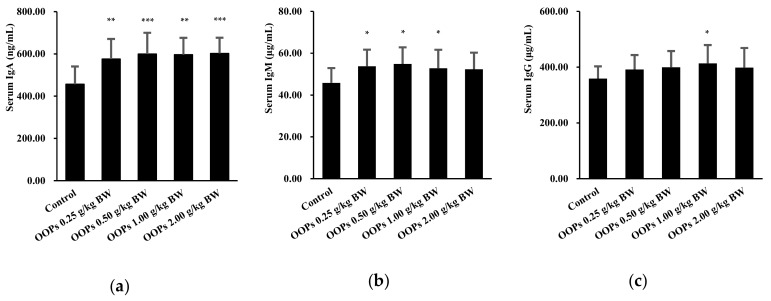
Effects of OOPs on serum immunoglobulin concentrations. (**a**) IgA concentrations in serum; (**b**) IgM concentrations in serum; (**c**) IgG concentrations in serum. Data are represented as mean ± SD, *n* = 10. * *p* < 0.05 vs. control group, ** *p* < 0.01 vs. control group, *** *p* < 0.001 vs. control group.

To investigate the mechanism via which OOPs regulated humoral immunity, serum immunoglobulin levels were evaluated. As shown in Figure 5a, serum IgA levels in all four OOPs groups showed significant increases (*p* < 0.01, *p* < 0.001, *p* < 0.01, and *p* < 0.001). Moreover, mice treated with OOPs at doses of 0.25, 0.5, and 1.0 g/kg BW showed significant increases in serum IgM concentrations compared with that in the control group (*p* < 0.05) with an increasing trend in 2.0 g/kg BW group (*p* = 0.062, Figure 5b). As showed in Fig. 5c, serum IgG concentrations were elevated in the OOPs 1.0 g/kg BW groups (*p* < 0.05) with increased trending in 0.5 (*p* = 0.135) and 2.0 g/kg BW (*p* = 0.149) groups.

## 4. Discussion

Oats are one of the most widely recommended grains with the highest protein concentration among cereals. A number of studies have focused on oat β-glucan, while few have focused on oat proteins let alone oat oligopeptides. Oat protein is known for its positive effects on celiac disease, blood glucose, and cholesterol metabolism, and for its antioxidant and anti-fatigue effects [16,23,24,25,26]. On the other hand, food-derived peptides have been considered as one of the most effective alternatives to drugs as immunomodulators [27]. Therefore, OOPs, mainly composed of small molecule oligopeptides derived from oat (*Avena nuda* L.) bran, can also be potential immunomodulators to enhance adaptive and innate immunities which form the immune system in vertebrates. Adaptive immunity is highly specific to antigens, including cell-mediated and humoral (antibody-mediated) immunity. Innate immunity provides non-specific protection, acting as the front line of defense through anatomical, physiological, cellular, and inflammatory components. Among immune cells such as dendritic cells, macrophages, NK cells, and neutrophils [22,28], macrophages and polymorphonuclear leukocytes specialize in phagocytosis, while NK cells defend against virus-infected and tumor cells. Since the mechanism through which food-derived bioactive peptides regulate the immune system remains uncertain, we evaluated the immunomodulatory effects of OOPs via four aspects including macrophage phagocytosis, NK cell activity, cell-mediated immunity, and humoral immunity with seven assays. The results of our study showed that OOPs treatments could improve adaptive immunity through improving delayed-type hypersensitivity, ConA-stimulated splenic lymphocyte proliferation, spleen IgM responses to SRBC, and HC_50_ levels, hence enhancing cell-mediated and humoral immunities, respectively. As for innate immunity, we found that OOPs could enhance the phagocytic capacity of macrophages and NK cell activity.

With further investigation, the immunomodulatory mechanism of OOPs may attribute to the regulation of lymphocyte function, cytokine secretion, and antibody production. To start with, since the key role of cell-mediated immunity is T lymphocytes (T cells, CD3^+^), consisting of helper T cells (Th cells, CD4^+^), cytotoxic T cells (Tc cells, CD8^+^), and regulatory T cells (Treg cells), subsets of T lymphocyte were analyzed. Our study found that the percentages of CD3^+^ and CD4^+^ T cells were significantly increased after OOPs treatment, indicating improvement in T cell quantity and proportion especially in Th cells, which can secrete cytokines and mediate cellular immune responses with antigen presenting cells (APCs), in accordance with our adaptive immunity test results.

To be specific, CD4^+^ T cells consist of T helper type 1 (Th1) cells, which induce cell-mediated immune responses via producing inflammatory cytokines of IL-2, IFN-γ, GM-CSF, and TNF-α, and T helper type 2 (Th2) cells, which induce humoral responses by secreting cytokines of IL-4, IL-5, and IL-10 [29]. With further investigation in the cytokine profile, we found increases in Th1 secreted cytokines of IL-2, IFN-γ, GM-CSF, and TNF-α in the OOPs groups. The elevated levels of these cytokines led to the improvement of Th1 cell differentiation and growth, secretion of IL-1 and IL-6, elevated functions of B cells, NK cells, and macrophagocytes as well as the negative accommodation of Th2 cells [30], partly explaining the positive effects of OOPs on the enhancement of cell-mediated immunity assessments including DTH and ConA-stimulated splenic lymphocyte proliferation, and contributing to the improvement of NK cell activity and macrophagocyte function. Moreover, elevated levels of IL-12 (mainly secreted by dendritic cells) in OOPs 1.0 and 2.0 g/kg BW groups implied the enhancement of APC function, which is a vital promoting factor in Th1 cell immune responses and an inhibitor of Th2. As for Th2 cell-secreted cytokines, we also found gradually elevated levels of IL-10, a major immunosuppressive factor, along with increasing doses of OOPs treatments. On the other hand, IL-2 can also improve Treg cell function, which is an inhibitor of CD4^+^ T cells. Due to the interactions between Th1, Th2 cells and the cytokines they produced, the inhibition of IL-10 and Treg cells in Th1 cell function and secretions of IL-2, IFN-γ, GM-CSF, and TNF-α were likely to result in an unclear dose-effect relation of Th1-secreted cytokines. Possibly along with the inhibition effects of IL-12 and IFN-γ in Th2 cells, the significant changes in IL-4 and IL-5 weren’t observed. In addition, increases in mononuclear macrophage-secreted cytokines such as IL-1α and IL-6 were also found in all four OOPs groups, leading to activated NK cells and enhanced macrophage function, which were in accordance with NK cell activity and macrophagocyte phagocytosis assay results. These results may also attribute to the improvement of macrophagocytes acting as APCs. By processing and presenting antigens to lymphocytes, macrophagocytes can assist T and B lymphocytes in recognizing antigens and regulating immune responses.

On the other hand, humoral immunity involves B lymphocytes (B cell) as a significant part of adaptive immune responses, producing antibodies, binding to antigens, and labeling them for macrophage destruction. Antibodies are the main effectors in humoral immune responses. When the host is under pathogen attack, circulatory IgM and antimicrobial peptides are constitutively expressed as low-cost defense effectors. IgA plays an important role in mucosal immunity by transcytosis across epithelial cells in frontline immunity [31]. When these responses above are not sufficient, IgG as the richest and most long-lasting antibody in serum is induced, which is crucial in monocyte-macrophage phagocytosis to defend against infections [32]. A previous study has shown that protein hydrolysates from rohu (Labeo rohita) egg (roe) significantly enhanced humoral immune responses (IgA) [33]. We more notably observed that OOPs treatment induced major rises in serum IgA, IgM, and IgG concentrations, contributing to humoral immunity, macrophage phagocytosis, and NK cell activity improvements. These can be attributed to antibody functions of IgA, IgM, and IgG as well as the interaction between innate and adaptive immune systems. Combined with the results of cytokine secretions, improvements effects of OOPs on humoral immunity are more likely via increasing antibody production rather than cytokine secretion stimulation. In the future, specific effective peptide segments and sequences can be investigated, as well as the possible content changes after intestinal digestion to further elucidate the biochemical mechanisms of immune enhancement effects of OOPs. Meanwhile, the immunoregulation effects of OOPs under special conditions such as autoimmunity and immunosuppression are also worth exploring.

## 5. Conclusions

Our study clearly demonstrated that OOPs had potentials to enhance innate and adaptive immune responses via improvements in macrophage phagocytosis, NK cell activity, cell-mediated immunity, and humoral immunity. Those effects are most likely due to the stimulation of Th cells, as well as cytokine secretion and immunoglobulin production, indicating that dietary OOP could be a promising immunomodulator candidate especially at doses from 0.25 g to 2.0 g/kg BW.

## Figures and Tables

**Table 1:** Amino acid composition of OOPs.

Amino Acid	Composition of OOPs (g/100g)
Alanine	4.43
Arginine	4.08
Aspartate	5.62
Cysteine	1.89
Glutamate	13.61
Glycine	4.24
Histidine	1.46
Isoleucine	2.47
Leucine	4.66
Lysine	2.82
Methionine	1.26
Phenylalanine	3.30
Proline	3.67
Serine	3.45
Threonine	2.73
Tyrosine	2.72
Valine	3.72

**Table 2:** Effects of OOPs on body weight and immune organ index of mice.

Groups	Initial Body Weight (g, *N* = 40)	Final Body Weight (g, *N* = 40)	Index of Spleen (mg/g, *N* = 10)	Index of Thymus (mg/g, *N* = 10)
Control	18.43 ± 0.94	19.56 ± 0.82	3.65 ± 0.30	1.87 ± 0.27
OOPs 0.25 g/kg BW	19.41 ± 1.11	20.33 ± 0.75	3.48 ± 0.58	2.05 ± 0.52
OOPs 0.5 g/kg BW	19.50 ± 1.13	19.72 ± 1.27	3.67 ± 0.35	2.09 ± 0.59
OOPs 1.0 g/kg BW	18.78 ± 0.72	20.08 ± 1.17	3.65 ± 0.42	2.27 ± 0.50
OOPs 2.0 g/kg BW	19.23 ± 0.95	20.56 ± 1.21	3.52 ± 0.34	1.92 ± 0.50

The data are presented as mean ± SD.

**Table 3:** Effects of OOPs on splenic T lymphocyte subpopulations.

Groups.	CD3^+^ (%)	CD4^+^ CD8^−^ (%)	CD4^−^ CD8^+^ (%)	CD4^+^ CD8^−^/CD4^−^ CD8^+^ (%)	CD4^+^ CD25^+^ (%)
Control	40.50±4.69	58.77 ± 1.47	34.32 ± 2.64	1.72 ± 0.11	7.67 ± 1.05
OOPs 0.25 g/kg BW	42.51 ± 4.51	59.96 ± 1.04	34.18 ± 3.18	1.77 ± 0.18	7.91 ± 1.87
OOPs 0.5 g/kg BW	42.67 ± 4.73	59.9 ± 2.19	34.39 ± 4.32	1.77 ± 0.29	8.83 ± 1.54
OOPs 1.0 g/kg BW	45.33 ± 2.81 *	60.59 ± 1.29 **	33.22 ± 2.55	1.84 ± 0.18	8.69 ± 1.48
OOPs 2.0 g/kg BW	45.63 ± 3.95 **	60.68 ± 1.11 **	31.97 ± 2.73	1.91 ± 0.19	8.63 ± 1.33

The data are presented as mean ± SD, *n* = 10. * *p* < 0.05 vs. the control group, ** *p* < 0.01 vs. the control group.
